# Supplementary material for: Content-rich biological network constructed by mining PubMed abstracts
Source: BMC Bioinformatics. 2004 Oct 8;5:147. doi: 10.1186/1471-2105-5-147 (PMC528731; doi:10.1186/1471-2105-5-147)
Supplement: Additional File 2 — The original results of the above study (non-essential files are deleted to keep the file size under the limit set by BMC bioinformatics). [file 1471-2105-5-147-S2.bz2 › chilibotAdditionalFile2/dip50/401ID7539796E9997/html/CNTF_CNTFR.html]

 


 **CNTF** and **CNTFR** 
  
Found 96 abstracts in PubMed, retrieved 50.  
 

 What does Google say? 
 PDF only 
| .edu only 

---

**Interactive relationship** (e.g. stimulation, inhibition, etc)

**Inhibitory relationship**- The functional receptor complex of  **CNTF**  is composed of the  **CNTF**  receptor alpha  **CNTFR** , gp130 and the leukemia inhibitory factor receptor LIFR.  Ref: 11943154 FEBS Lett, 2002
- Mice lacking  **CNTFR**  alpha  [ **CNTFR** ]  die perinatally, perhaps indicating the existence of a second developmentally important  **CNTF**  like ligand.  Ref: 10812968 Pharm Acta Helv, 2000
- Ciliary neurotrophic factor  [ **CNTF** ]   **CNTF**  drives the sequential assembly of a receptor complex containing the ligand specific alpha receptor subunit  **CNTFR**  and the signal transducing beta subunits gp130 and leukemia inhibitory factor receptor beta LIFR.  Ref: 9287306 J Biol Chem, 1997
- Human  **CNTF**  elicits cellular responses by induction of a receptor complex consisting of the  **CNTF**  a receptor  **CNTFR** , which is not involved in signal transduction, and the b receptors gp130 and leukaemia inhibitory factor receptor LIFR.  Ref: 12517918 J Biol Chem< MedlineTA>J Biol Chem, 1997
- Human  **CNTF**  elicits cellular responses by induction of a receptor complex consisting of the  **CNTF**  alpha receptor  **CNTFR** , which is not involved in signal transduction, and the beta receptors gp130 and leukemia inhibitory factor receptor LIFR.  Ref: 12643274 J Biol Chem, 2003
- The functional receptor complex of ciliary neurotrophic factor  [ **CNTF** ]   **CNTF** , a member of the gp130 family of cytokines, is composed of  **CNTF** , the  **CNTF**  receptor alpha  **CNTFR** , gp130 and the leukemia inhibitory factor receptor LIFR.  Ref: 12707266 J Biol Chem< MedlineTA>J Biol Chem, 2003
- For signal transduction,  **CNTF**  requires a receptor complex, composed of the IL 6 signal transducing molecule gp130, leukemia inhibitory factor receptor LIFR beta, and  **CNTFR**  alpha  [ **CNTFR** ] .  Ref: 11464862 Med Pediatr Oncol, 2001
- **CNTF**  functional responses are mediated through a tripartite membrane receptor composed of two signalling receptor chains, gp130 and the leukaemia inhibitory factor receptor LIFR, associated with a non signalling  **CNTF**  binding receptor alpha component  **CNTFR** .  Ref: 11285233 EMBO J, 2001
**Neutral relationship**- These findings suggest a possible role of  **CNTF**  as a growth factor during renal tubular repair processes after I R injury and an autocrine or paracrine function of  **CNTF**  acting against  **CNTFR**  alpha  [ **CNTFR** ] .  Ref: 11274236 J Am Soc Nephrol, 2001
- Membrane anchoring of  **CNTFR**  was found to render the  **CNTF**  receptor complex relatively insensitive to changes in agonist affinity for either alpha or beta receptor subunits and to promote a more efficient interaction with a gp130 depleting antagonistic variant of  **CNTF** .  Ref: 9287306 J Biol Chem, 1997
- Engineering a  **CNTFR**  specific human  **CNTF**  variant may therefore be a prerequisite to improve the safety profile of  **CNTF** .  Ref: 12517918 J Biol Chem< MedlineTA>J Biol Chem, 1997
- Engineering a  **CNTFR**  specific human  **CNTF**  variant may therefore be a prerequisite to improving the safety profile of  **CNTF** .  Ref: 12643274 J Biol Chem, 2003
- These data suggest that both the gp130 CBD and the LIFR CBD1 interact with  **CNTFR**  in a similar way and provide insights into the nature of the  **CNTF**  receptor complex.  Ref: 12707266 J Biol Chem< MedlineTA>J Biol Chem, 2003
- Modelling studies of the interleukin 6 family member cardiotrophin like cytokine CLC revealed structural similarities with  **CNTF** , including the conservation of a site I domain involved in binding to  **CNTFR** .  Ref: 11285233 EMBO J, 2001
- We now demonstrate that the responsiveness of photoreceptor precursors to  **CNTF**  is confined to a brief phase between their final mitosis and their terminal differentiation owing to the temporally restricted expression of the  **CNTF**  receptor  **CNTFR**  alpha  [ **CNTFR** ] .  Ref: 9858267 J Neurobiol, 1998
- Furthermore,  **CNTF**  variants coadministered with sCNTFR alpha protected against LPS toxicity in a manner related to their affinity for the beta components of  **CNTFR** .  Ref: 9656141 J Inflamm, 1998
- These results demonstrate that after fimbria fornix transection  **CNTFR**  alpha  [ **CNTFR** ]  expression is transiently induced in axotomized, non cholinergic neurons of the medial septal nucleus, suggesting a postlesion function of locally supplied  **CNTF** .  Ref: 9104604 Eur J Neurosci, 1997

**Non-interactive relationship** (e.g. studied together, co-existance, homology, etc.)

- The mRNA levels of neuropoietic cytokines, ciliary neurotrophic factor  [ **CNTF** ]   **CNTF** , leukemia inhibitory factor LIF, and interleukin 6 IL 6, and their receptor components  **CNTFR**  alpha  [ **CNTFR** ] , LIFR beta, IL 6R alpha, and gp130 were examined in seventy six patients with various peripheral neuropathies to determine the extent of expression of these cytokines and receptors, and their relationship to nerve fiber pathology and cell infiltration in the diseased nerves.  Ref: 11358282 Neurochem Res, 2001
- We characterized the survival factor response of the normal mouse retina to mechanical injury by examining the expression of mRNAs for basic fibroblast growth factor bFGF, ciliary neurotrophic factor  [ **CNTF** ]   **CNTF** , and their receptors, FGF receptor 1 FGFR 1 and  **CNTF**  receptor alpha  **CNTFR**  alpha  [ **CNTFR** ] .  Ref: 9268592 Exp Eye Res, 1997
- The C174T polymorphism in the  **CNTF**  receptor  **CNTFR**  gene is associated with fat free mass in men and women.  Ref: 12807897 J Appl Physiol< MedlineTA>J Appl Physiol, 1997
- In addition, the expression of ciliary neurotrophic factor  [ **CNTF** ]  receptor  **CNTFR**  and leukemia inhibitory factor receptor LIFR mRNAs, but not of interleukin 6 receptor mRNA, was up regulated after systemic lipopolysaccharide treatment.  Ref: 12127095 Neuroscience, 2002
- To investigate this, the expression of  **CNTF**  and  **CNTF**  receptor alpha  **CNTFR**  alpha  [ **CNTFR** ]  was analyzed in the ischemic rat kidney.  Ref: 11274236 J Am Soc Nephrol, 2001
- Striking phenotypic differences between  **CNTF**  and  **CNTFR**  deficient mice suggest that  **CNTFR**  serves as a receptor for a second developmentally important ligand.  Ref: 11294841 J Biol Chem, 2001
- In this study, we provide the first demonstration that the membrane distal CBD CBD1 of LIFR associates in vitro with soluble  **CNTFR**  in the absence of  **CNTF** .  Ref: 11943154 FEBS Lett, 2002
- The specificity conferring alpha subunit of the  **CNTF**  complex  **CNTFR**  alpha  [ **CNTFR** ] , is extremely well conserved across species, and has a distribution localized predominantly to the nervous system and skeletal muscle.  Ref: 10812968 Pharm Acta Helv, 2000
- A hormone response element, CNTFRalpha NBRE 5 AAAGGTCA 3 has been identified in the fifth intron of the alpha component of ciliary neurotrophic factor  [ **CNTF** ]  receptor gene  **CNTFR**  15 for the human TR3 orphan receptor TR3.  Ref: 9798727 Endocrine, 1998
- In summary, differential regulation of  **CNTF**  and  **CNTFR**  alpha  [ **CNTFR** ]  mRNAs was noted in the ischemic cortex.  Ref: 9645962 Brain Res Mol Brain Res, 1998
- **CNTF**  receptor alpha  **CNTFR**  alpha  [ **CNTFR** ] , the specific component of the tripartite  **CNTF**  receptor system, was detectable by Northern blot analysis in 80% of the cases.  Ref: 10720204 J Neurooncol, 1999
- In the present study, we examined the temporal and spatial expression profiles of  **CNTF**  and  **CNTF**  receptor alpha  **CNTFR**  alpha  [ **CNTFR** ]  mRNAs in a focal cerebral ischemia model induced by transient occlusion of the right middle cerebral artery and both common carotid arteries.  Ref: 9645962 Brain Res Mol Brain Res, 1998
- The expression of  **CNTF**  and  **CNTFR**  alpha  [ **CNTFR** ]  was monitored by reverse transcription PCR, in situ hybridization, immunoblotting, immunohistochemistry, and electron microscopy.  Ref: 11274236 J Am Soc Nephrol, 2001
- This study reinforces the functional similarity between  **CNTF**  and the CLC CLF composite cytokine defining the second ligand for  **CNTFR** .  Ref: 11294841 J Biol Chem, 2001
- In contrast to that reported before for  **CNTF** , soluble  **CNTFR**  failed to promote the action CLC CLF, and an absolute requirement of the membrane form of  **CNTFR**  was required to generate a functional response to the composite cytokine.  Ref: 11294841 J Biol Chem, 2001
- The time course of  **CNTFR**  alpha  [ **CNTFR** ]  expression and the in vitro effects suggest that  **CNTF**  or a related molecule is required during early stages of rod differentiation, while differentiated rods depend on additional protective factors for survival.  Ref: 9858267 J Neurobiol, 1998
- Continuous treatment with Dex 10 8 mol L inhibited the endogenous production of IL 6, LIF, OSM, IL 11R, and OSMR, but had no detectable effect on the expression of IL 11, CT 1,  **CNTF** ,  **CNTFR** , LIFR, or gp130.  Ref: 12110437 Bone, 2002
- Expression of mRNAs for ciliary neurotrophic factor  [ **CNTF** ]   **CNTF** , leukemia inhibitory factor LIF, interleukin 6 IL 6, and their receptors  **CNTFR**  alpha  [ **CNTFR** ] , LIFR beta, IL 6R alpha, and gp130 in human peripheral neuropathies.  Ref: 11358282 Neurochem Res, 2001
- The ciliary neurotrophic factor  [ **CNTF** ]  and its receptor,  **CNTFR**  alpha  [ **CNTFR** ] .  Ref: 10812968 Pharm Acta Helv, 2000
- Striking phenotypic differences between  **CNTF**  and  **CNTFR**  deficient mice suggest that  **CNTFR**  serves as a receptor for a second, developmentally important ligand.  Ref: 10966616 Nat Neurosci, 2000
- In contrast, inactivation of the  **CNTF**  gene leads only to a slight muscle weakness, mainly during adulthood, suggesting that  **CNTFR**  binds to a second ligand that is important for development.  Ref: 11285233 EMBO J, 2001
- Differential regulation of ciliary neurotrophic factor  [ **CNTF** ]   **CNTF**  and  **CNTF**  receptor alpha  **CNTFR**  alpha  [ **CNTFR** ]  expression following focal cerebral ischemia.  Ref: 9645962 Brain Res Mol Brain Res, 1998
- To investigate how this functional complexity relates to the regulation of  **CNTF**  receptor alpha  **CNTFR**  alpha  [ **CNTFR** ]  expression, we have studied the developmental expression of the receptor protein in chick retina by using immunocytochemistry.  Ref: 9766402 J Comp Neurol, 1998
- Previously, we reported that the nuclear orphan receptor TR4 could induce transcriptional activity via the 5th intron of the ciliary neurotrophic factor  [ **CNTF** ]   **CNTF**  alpha receptor gene  **CNTFR**  I5.  Ref: 9694834 J Biol Chem, 1998
- Following 1 week of reperfusion, although no marked changes was observed in the level of  **CNTFR**  alpha  [ **CNTFR** ]  mRNA in the area immediately surrounding the necrosis region where the reactive astrocytes were noted, a striking increase in the  **CNTF**  mRNA signal was noted.  Ref: 9645962 Brain Res Mol Brain Res, 1998
- We showed that ciliary neurotrophic factor  [ **CNTF** ]  receptor  **CNTFR**  gene expression was higher in muscle biopsy tissue from severely affected MG patients regardless of anti nAChR antibody titer.  Ref: 11694333 J Neuroimmunol, 2001
- Signal transduction by  **CNTF**  requires that it bind first to  **CNTFR**  alpha  [ **CNTFR** ] , permitting the recruitment of gp130 and LIFR beta, forming a tripartite receptor complex.  Ref: 10812968 Pharm Acta Helv, 2000
- The expression of  **CNTFR**  alpha  [ **CNTFR** ]  mRNA was also increased after I R injury, and its location and expression patterns were similar to the expression of  **CNTF** .  Ref: 11274236 J Am Soc Nephrol, 2001
- Differential temporal expression of mRNAs for ciliary neurotrophic factor  [ **CNTF** ]   **CNTF** , leukemia inhibitory factor LIF, interleukin 6 IL 6, and their receptors  **CNTFR**  alpha  [ **CNTFR** ] , LIFR beta, IL 6R alpha and gp130 in injured peripheral nerves.  Ref: 9630704 Brain Res, 1998
- We previously reported that the membrane distal cytokine binding domain CBD1 of LIFR could interact in vitro with soluble  **CNTFR**  sCNTFR in the absence of  **CNTF** .  Ref: 12707266 J Biol Chem< MedlineTA>J Biol Chem, 1998
- **CNTF**  mRNA, but not  **CNTFR**  alpha  [ **CNTFR** ]  mRNA, had a marked increase in the area immediately adjacent to the necrosis.  Ref: 9645962 Brain Res Mol Brain Res, 1998
- Two new types of  **CNTF**  variants, characterized by weakened interactions with either  **CNTFR**  or both LIFR and gp130, were developed, and the biological activities of these and other mutants were determined in non neuronal versus neuronal cells, as well as in non neuronal cells transfected with an expression vector for  **CNTFR** .  Ref: 9287306 J Biol Chem, 1997
- **CNTFR**  alpha  [ **CNTFR** ]  alone or in combination with  **CNTF**  promotes macrophage chemotaxis in vitro.  Ref: 11162290 Neuropeptides, 2000
- We performed gene screening of the ciliary neurotrophic factor  [ **CNTF** ]  receptor  **CNTFR**  gene and genotyped three newly identified polymorphisms C 1703T in the 5 promoter region, T1069A in intron 5, and C174T in exon 9.  Ref: 12807897 J Appl Physiol< MedlineTA>J Appl Physiol, 2000
- Given the restricted expression domain of the  **CNTF**  receptor alpha  **CNTFR**  in the developing forebrain germinal zone and adult forebrain periventricular area, we have examined the putative role of  **CNTFR**  LIFR gp130 mediated signaling in regulating forebrain neural stem cell fate in vivo and in vitro.  Ref: 11567054 J Neurosci, 2001
- Using non radioactive in situ hybridization we investigated the effect of fimbria fornix transection on the expression of ciliary neurotrophic factor  [ **CNTF** ]  receptor alpha  **CNTFR**  alpha  [ **CNTFR** ]  mRNA in axotomized septohippocampal neurons of the rat septal complex.  Ref: 9104604 Eur J Neurosci, 1997
